# The impact of leadership hubs on the uptake of evidence-informed nursing practices and workplace policies for HIV care: a quasi-experimental study in Jamaica, Kenya, Uganda and South Africa

**DOI:** 10.1186/s13012-016-0478-3

**Published:** 2016-08-03

**Authors:** Nancy Edwards, Dan Kaseje, Eulalia Kahwa, Hester C. Klopper, Judy Mill, June Webber, Susan Roelofs, Jean Harrowing

**Affiliations:** 1 School of Nursing, Faculty of Health Sciences, University of Ottawa, Ottawa, Canada; 2 Great Lakes University of Kisumu, Kisumu, Kenya; 3 School of Nursing, University of West Indies, Mona, Kingston Jamaica; 4 Rectorate, Stellenbosch University, Cape Town, South Africa; 5 Faculty of Nursing, University of Alberta, Edmonton, Canada; 6 Coady International Institute, St. Francis Xavier University, Antigonish, Canada; 7 Faculty of Health Sciences, University of Lethbridge, Lethbridge, Canada

**Keywords:** Nurses, Leadership, Participatory action research, HIV, Low- and middle-income countries, Capacity building, Health system strengthening, Evidence-informed clinical practice, Workplace policies, Quality assurance, Stigma

## Abstract

**Background:**

The enormous impact of HIV on communities and health services in Sub-Saharan Africa and the Caribbean has especially affected nurses, who comprise the largest proportion of the health workforce in low- and middle-income countries (LMICs). Strengthening action-based leadership for and by nurses is a means to improve the uptake of evidence-informed practices for HIV care.

**Methods:**

A prospective quasi-experimental study in Jamaica, Kenya, Uganda and South Africa examined the impact of establishing multi-stakeholder leadership hubs on evidence-informed HIV care practices. Hub members were engaged through a participatory action research (PAR) approach. Three intervention districts were purposefully selected in each country, and three control districts were chosen in Jamaica, Kenya and Uganda. WHO level 3, 4 and 5 health care institutions and their employed nurses were randomly sampled. Self-administered, validated instruments measured clinical practices (reports of self and peers), quality assurance, work place policies and stigma at baseline and follow-up. Standardised average scores ranging from 0 to 1 were computed for clinical practices, quality assurance and work place policies. Stigma scores were summarised as 0 (no reports) versus 1 (one or more reports). Pre-post differences in outcomes between intervention and control groups were compared using the Mantel Haenszel chi-square for dichotomised stigma scores, and independent *t* tests for other measures. For South Africa, which had no control group, pre-post differences were compared using a Pearson chi-square and independent *t* test. Multivariate analysis was completed for Jamaica and Kenya. Hub members in all countries self-assessed changes in their capacity at follow-up; these were examined using a paired *t* test.

**Results:**

Response rates among health care institutions were 90.2 and 80.4 % at baseline and follow-up, respectively. Results were mixed. There were small but statistically significant pre-post, intervention versus control district improvements in workplace policies and quality assurance in Jamaica, but these were primarily due to a decline in scores in the control group. There were modest improvements in clinical practices, workplace policies and quality assurance in South Africa (pre-post) (clinical practices of self—pre 0.67 (95 % CI, 0.62, 0.72) versus post 0.78 (95 % CI, 0.73–0.82), *p* = 0.002; workplace policies—pre 0.82 (95 % CI, 0.70, 0.85) versus post 0.87 (95 % CI, 0.84, 0.90), *p* = 0.001; quality assurance—pre 0.72 (95 % CI, 0.67, 0.77) versus post 0.84 (95 % CI, 0.80, 0.88)). There were statistically significant improvements in scores for nurses stigmatising patients (Jamaica reports of not stigmatising—pre-post intervention 33.9 versus 62.4 %, pre-post control 54.7 versus 64.4 %, *p* = 0.002—and Kenya pre-post intervention 35 versus 51.6 %, pre-post control 34.2 versus 47.8 %, *p* = 0.006) and for nurses being stigmatised (Kenya reports of no stigmatisation—pre-post intervention 23 versus 37.3 %, pre-post control 15.4 versus 27 %, *p* = 0.004). Multivariate results for Kenya and Jamaica were non-significant. Twelve hubs were established; 11 were active at follow-up. Hub members (*n* = 34) reported significant improvements in their capacity to address care gaps.

**Conclusions:**

Leadership hubs, comprising nurses and other stakeholders committed to change and provided with capacity building can collectively identify issues and act on strategies that may improve practice and policy. Overall, hubs did not provide the necessary force to improve the uptake of evidence-informed HIV care in their districts. If hubs are to succeed, they must be integrated within district health authorities and become part of formal, legal organisations that can regularise and sustain them.

**Electronic supplementary material:**

The online version of this article (doi:10.1186/s13012-016-0478-3) contains supplementary material, which is available to authorized users.

## Background

HIV has had an enormous impact on the health of communities and on models of service delivery [1–5] particularly in sub-Saharan Africa, and more recently in the Caribbean. While all health providers have had to deal with this epidemic, nurses and midwives^1^ who deliver direct client care and comprise the largest proportion of the health workforce in low- and middle- income countries (LMICs) have been especially affected. Severe nursing shortages and unrealistically low nurse-to-patient care ratios are entwined with the impact of stigmatisation, workplace safety and health service delivery in HIV prevalent settings [5–13]. These have all contributed to significant gaps in HIV care in LMICs [14–16].

Efforts to address these gaps have been targeted at different facets of the problem. Extensive professional development training and mentoring programmes for nurses have focused on clinical skills and scope of practice [17]. These have included strategies to reduce HIV stigma [12, 18], to improve patient counselling on adherence to anti-retroviral therapy (ART) [19], to enhance voluntary testing and counselling for HIV testing [19, 20] and to increase the update of universal precautions to protect both patients and nurses [21, 22].

Human resource constraints have been addressed at national and district levels with a focus on task sharing with nurses [23–29] and task shifting to community health workers (CHWs) [23–28, 30]. Integrated care models [31–39] have been introduced to improve appropriate referrals, ART initiation, coverage and retention in care. Quality improvement approaches for better care and workplace policies and programmes have been tested such as cascade and systems analyses [15, 40–46]. Achieving the full potential of these approaches, however, requires leadership capacity development for nurses to fully engage in enabling change [47] and to address issues such as navigating professional turf, providing supportive supervision for CHWs [48–50], and shifting the organisational context for care delivery [51].

Utilising the technical knowledge and clinical experiences of nurses in the formulation of related guidelines and policies by senior decision-makers and policy-makers [52–58] also requires nursing leadership. However, many of the leadership interventions to involve nurses in developing and implementing evidence-based guidelines have been undertaken in higher-income countries [59–62]. There is a paucity of studies examining ways to strengthen nursing leadership for improvements in the uptake of evidence-informed HIV care in LMICs [24, 63, 64]. Furthermore, much of the research on building leadership capacity has focused on concentrated, short-term leadership training programmes either targeted directly at nurses [65–67] or aimed at those holding management positions [68]. As Daire et al. [63] pointed out, there is an abundance of training that focuses on the cognitive aspects of leadership rather than on the action component. If nurses in LMICs are to play a more substantial role in reorienting HIV care strategies and workplace conditions to be more evidence-informed, this requires action-based leadership: nurses’ engagement in and capacity to lead, implement and sustain HIV care improvements [69–71]. This study focused on strengthening the leadership and policy engagement capacity of nurses to address gaps in HIV care and workplace policies in all health facilities in their health districts.

### Overview of study

The primary objective of this quasi-experimental study was to determine the impact of establishing leadership hubs on HIV care by nurses. We engaged hub members in a participatory action research (PAR) process to improve the uptake of evidence-informed nursing care practices and workplace policies for HIV. This study was undertaken in four countries (Jamaica, Kenya, South Africa and Uganda) between 2008 and 2012, as part of a larger programme^2^ of research and capacity building [72–74]. The main quantitative study findings are described in this paper. Qualitative results are published elsewhere [9, 56, 75–77]. This study was carried out with support from the Global Health Research Initiative (GHRI), a collaborative research funding partnership of the Canadian Institutes of Health Research, the Canadian International Development Agency, Health Canada, the International Development Research Centre and the Public Health Agency of Canada.

## Methods

### Leadership hub intervention

District-level^3^ leadership hubs were established in each country with the aim of stimulating district-wide health improvements in HIV care. The theory of change underlying the study was as follows:

1. Bringing together stakeholders from different system levels to form a district leadership hub; and
2. Providing hub members with training on research and evaluation, policy engagement and leadership; and
3. Using a participatory action research approach with hubs as they reflect on research findings from their districts about HIV care and policies;
4. Will strengthen hub members’ individual agency and collective capacity to plan, develop, implement and monitor district-level, evidence-informed change strategies;
5. And will thereby improve the delivery of evidence-informed HIV care by nurses and strengthen supporting policies in district health facilities.

Indicators related to our theory of change are described in Table 1.

**Table 1:** Theory of change and related indicators

Theory of change	Indicators
Bring together stakeholders from different system levels to form a district leadership hub	• # of hubs established and sustained to end of project
	• # of hub meetings held
	• Turnover rate of hub members
Provide hub members with training on research and evaluation, policy engagement and leadership	• Core slate of seven hub training workshops provided for hub members
	• Completion of professional exchange visits by hubs
	• Mentorship and training provided as hubs develop action plans and evaluation projects
Use a participatory action research approach with hubs as they reflect on research findings from their districts about HIV care and policies	• Action plans and evaluation projects reflect gaps identified through research
	• Country-specific research findings (quantitative and qualitative) are shared with hub members
Strengthen hub members’ individual agency and collective capacity to plan, develop, implement and monitor district-level, evidence-informed change strategies	• Hubs develop and implement action plans
	• Hub evaluation projects successfully completed
	• Hubs disseminate evaluation project findings to their institutions
	• Hub members self-assessment of capacity improvements in:
	°Leadership and team skills to improve health
	°Valuing policy relevance and access
	°Disseminating research findings
	°Appraising evidence and identifying gaps
	°Initiating and undertaking evaluation
	°Communicating with decision-makers
	°Valuing contributions from people in different roles and at different levels of the health system
Improve delivery of evidence-informed HIV care by nurses and strengthen supporting policies in district health facilities	• Pre/post, intervention/control findings related to stigma, clinical practices and workplace policies

Although Danschroder’s Consolidated Framework for Implementation Research [78] had not been published when we designed the intervention for this study, we drew heavily on some of the literature, which informed this framework, in developing the overall leadership hub approach and specific training modules. Our focus on change within organisations as well as at the district level reflects an often neglected facet of change (the interface between inner and outer organisational contexts). Our intervention was consistent with key elements of Danschroder’s framework including adapting the intervention to the local context, while retaining core components; building agency of the hub members to extend and use their affiliations and power for positive change; and using an active change process aimed at both individuals and organisations.

We had several inclusion criteria specific to the composition of each hub (see Table 2). We aimed to have every leadership hub composed of members from four stakeholder groups— direct care nurses and nurse managers; researchers; decision-makers; and community representatives. This mixed composition was intended to extend the vertical networks (across system layers) of the nurses who joined the hubs and to activate district-level change by stimulating horizontal collaboration among hub members. Leadership hubs were designed to act as the lever for change, or enabling mechanism, that would transform enhanced capacity into action leading to policy and practice change [72, 75, 77]. This hub intervention targeted improvements in policies and patient care within health care institutions as orchestrated through a district-level change process.

**Table 2:** Inclusion criteria for leadership hubs

Criteria for hub composition	• Each leadership hub will have six to nine members:
	Members of each hub will be drawn from different levels of authority and responsibility within the health system, from different disciplines and from four key stakeholder groups within each of the intervention districts:
	▪ Registered nurses, registered midwives, enrolled nurses and nurse managers working in hospitals or communities
	▪ Researchers: junior, intermediate or senior nurse researchers
	▪ Decision-makers: from the Ministry of Health; local representatives from nursing or other health professional and regulatory bodies and unions
	▪ Community representatives: from community groups active on HIV issues (e.g. people living with HIV, grandmothers looking after AIDS-orphaned children, women’s groups)
	• Aim to have one person on each hub who is a person living with HIV or AIDS
Criteria for individual hub members	• Country nationals
	• Lived or worked in the intervention district for some time
	• Intend to reside in intervention district for the duration of the project
	• Involved in committees or work related to HIV care, policies and/or programmes
	• Willing to commit to involvement in a leadership hub for the duration of the project

Leadership hub members were purposively recruited and selected by country research teams (country programme director and research staff), in consultation with the national advisory committee (with the exception of South Africa where there was no national advisory committee)

One hub was established in each intervention district. We aimed for six to nine members per hub, with one person on each hub being a person living with HIV. Hub members were recruited by the country research team who approached individuals or health care organisational leads and consulted with national advisory committees, asking them to identify potential participants. Inclusion criteria for individual hub members are shown in Table 2. Hub membership was voluntary and unpaid. National advisory committees, set up specifically for the research programme, were intended to inform hub activities. Leaders of professional nursing associations, nursing councils, policy-makers and experienced academics were invited to participate on these committees.

**Fig. 1:**
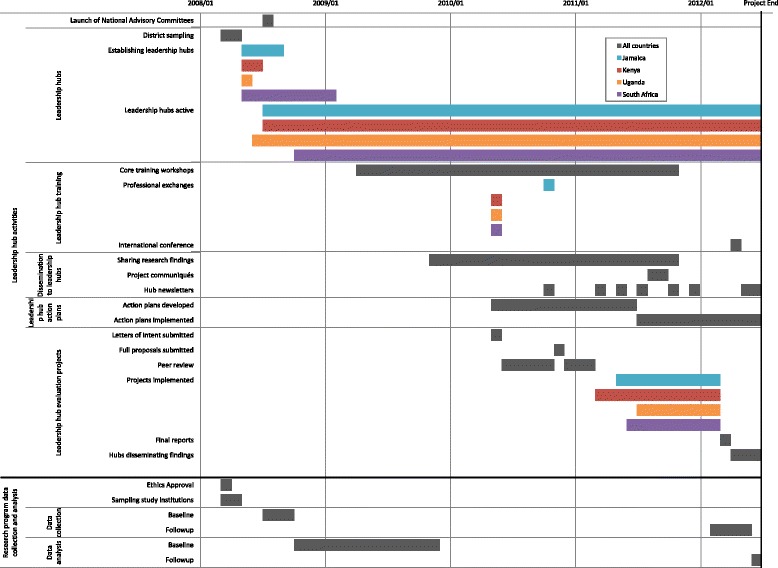
Chronology of the leadership hub intervention and research programme data collection/analysis

Standard elements of the hub intervention, implemented across all four countries, are shown in Table 3. A chronology of the hub intervention and research programme data collection/analysis is illustrated in Fig. 1. A common slate of training workshops (seven) was delivered to hubs over 3 years to build skills in using research evidence to identify gaps in clinical practice and workplace policies for their districts, using evidence generated by the research programme. The training approach was consistent with PAR processes [79] and designed to improve hubs’ abilities to identify, question and take action on setting-specific health system issues, thereby empowering members as change agents [76, 77]. Through the workshops, we addressed four aspects of capacity: ability (knowledge and skills); resources (financial, material, logistics); authority (control, voice, participation); and responsibility (accountability, monitoring). Interactive discussions during the workshops and the use of local examples tailored the content to study districts in each country. Midway into the second year of each hub's life, members were provided with country-specific baseline findings from our surveys of evidence-informed clinical practices and workplace policies and qualitative findings about the impact of HIV on nurses and their clinical practice. In-country research assistants (RAs) encouraged hub members to interrogate and use findings to identify priority activities they could undertake and stakeholders they should engage. Hubs were trained to develop and implement action plans to address gaps identified in workplace or district policies and practices related to HIV care. Commencing in year 3, a bi-monthly project newsletter was distributed to hubs to encourage information sharing. Three country-specific communiques were prepared to share findings and implications with institutional and district-level decision-makers.

**Table 3:** Leadership hub model—standard intervention elements for development and implementation activities

Intervention development activities
• Training sessions about leadership hubs delivered to country research staff.
• Specific training topics and objectives identified by research team in consultation with leadership hub members.
• Training materials prepared for interactive workshops. These were developed and/or adapted from training materials from other sources, by research team members with expertise pertinent to topic.
• Some workshop materials piloted with research assistants of project.
• Format for hub action plan reporting developed by research team members.
• Requirements and guidance document for developing and implementing evaluation projects developed by research team members.
• Objectives, format and processes for sharing and discussing quantitative and qualitative findings from research developed by research team and research assistants.
• Format for newsletter developed by research assistant in consultation with hub members.
• Format for district level communique and international newsletter developed by research team members in consultation with hub members who advised on what findings would be most pertinent to their managers.
Intervention implementation activities
• Leadership hubs established in three intervention districts in each of four LMIC countries.
• Slate of seven core training workshops delivered to hubs over 3 years to build capacity in research, policy engagement and leadership.
• Regular, ongoing mentoring of hubs (via telephone, field visits and joint hub meetings) provided by country research staff regarding development and implementation of action plans and evaluation projects.
• Research findings shared with hubs to stimulate critical reflection and action using project data from study districts. Quantitative research findings shared included stigma, and nursing clinical practices and policies related to HIV care. Qualitative research findings shared focused on the impact of HIV on nursing workforce.
• Three project communiques, produced for hubs, presented country-specific research findings on common topics (nursing clinical practices and policies, and HIV-related stigma).
• Eight issues of an international hub newsletter produced for hubs (documented project research findings, hub evaluation project results, profiles of hubs) to encourage sharing and exchange among hubs.
• District health action plans created by each hub addressed gaps identified through research findings.
• Evaluation projects (funded through small grants from our research programme) were written, peer reviewed, revised and implemented; findings were analysed (with assistance of research assistants) and disseminated to stakeholders by all active hubs.
• Hubs participated in professional exchange visits (e.g. meetings with national and international agencies involved in HIV; exchange visits between hubs in two countries; participation in international hub teleconferences to discuss nursing strategies).
• Leaders of all active hubs participated in and presented findings from study at an international conference (World Congress on Public Health held in Ethiopia).

In years 4 and 5, we offered each hub a small grant (maximum $1000 per project) to plan and undertake an evaluation project examining local district-level gaps in health policy or practice related to HIV nursing care. Country research teams provided mentorship and a peer review process, led by NE, provided further input to strengthen each evaluation project. Their letters of intent and subsequent proposals were formally reviewed by the research team (team members from each study country and Canada) and interns participating in the Jamaica international research internship for health system researchers (a capacity building initiative for junior researchers, also funded by the research programme). Hubs received extensive written feedback from the peer review committee, and RAs and hubs received training in how to respond to peer reviewers. Hub members obtained institutional letters of permission to undertake their projects.

Hubs received regular mentoring throughout the project at all country sites. Country RAs worked closely with hubs, supporting them through field visits and telephone calls. They coordinated hub meetings, facilitated hub training sessions and mentored hubs through the process of developing action plans, and implementing and disseminating evaluation projects. Two international teleconferences facilitated by the Canadian programme manager brought together hubs from all countries for discussions about their evaluation projects, action plans and nursing strategies for common workplace and policy issues. The Canadian programme manager mentored country RAs throughout this process by email and Skype, encouraged peer-to-peer learning and led three face-to-face refresher training sessions later in the project.

### Study context

The study was conducted in countries located within the two regions most affected by HIV. In sub-Saharan Africa, which has 71 % of all HIV cases worldwide, an estimated 25.8 million people are living with the disease, a prevalence of 2.8 % of the total population [80, 81]. South Africa has the highest HIV prevalence in the region, at 11.2 % of the total population, while Kenya and Uganda have prevalence rates of 3.8 and 3.7 %, respectively [80]. The Caribbean is the second most affected region of the world with an overall HIV prevalence of 1.1 % (0.9–1.2 %), and 250,000 adults and children in the region living with the disease [81]. In Jamaica, the HIV prevalence rate is 1.2 %.

### Country-specific adaptations to the intervention

Since the hub intervention was intended to be implemented using a PAR process, it was essential that the intervention was appropriately tailored to diverse settings. Certain intervention components were intended to be adapted by each country to increase relevance to their context, an important consideration particularly in low-resource settings and given that implementation contexts and priority health issues varied greatly across countries. The proportion of hub members to be drawn from each of the four stakeholder groups was not a pre-set requirement. Country research teams identified members based on receptivity to engagement, availability to commit for 4 years and the local context, and built on existing institutional links between the workplaces of country project leads and other organisations. While most hub members worked at the district level, Uganda (which had one hub based in the national capital) had a hub member who was a national member of parliament. While the aim was to have representation of an HIV-positive person on each hub, this depended on the self-disclosure by potential members of their HIV status; voluntary self-disclosure of HIV status was ethically essential and made it impossible to mandate a required number of HIV-positive members. The number of self-identified HIV-positive hub members ranged from one member per hub (Jamaica) to one member per country (Kenya, Uganda and South Africa).

Flexibility was deliberately built into other intervention elements to allow country research teams to tailor to setting. While all intervention activities were undertaken in the same 4-year period, the sequencing of some activities varied by country, such as the dates that all three hubs in a given country were established (Uganda was first to achieve this, in May 2008; South Africa was last, in February 2009 due to challenges in hub member recruitment). The timing of training workshops depended on participant availability, while the timing of grant approval and rollout of hub projects depended on hub turnaround time in response to sometimes extensive peer review comments.

Communiques shared with hubs with research findings from the programme presented country-specific findings on the same topics: nursing clinical practices, HIV stigma and human resources management; Jamaica developed an additional communique (HIV training and policy). Countries tailored workshop training materials using setting-specific examples and added workshops (one to three workshops per country) based on hub learning needs and interests. The focus for hub action plans and hub evaluation projects was chosen by each hub, within the guidelines set by the project stipulating that the topic had to emerge from gaps identified by the programme’s research findings and be responsive to the local health situation. Hubs could choose whether to undertake their projects on their own or jointly with another hub; in two countries (Kenya and South Africa), two hubs chose to work jointly on a project.

There were also unplanned divergences in implementation. This included the lack of a functioning national advisory committee in one country (the committee in South Africa ceased shortly after inception). In Kenya and Uganda, several hub members responded to nationally mandated redeployment of health personnel by maintaining their commitments, albeit at a distance, to their original hub (while they moved to new workplaces outside of their original intervention district, none had been redeployed to control districts). While all three hubs per country were sustained in three of the study countries, one hub (South Africa) ceased to function before the end of the project.

### Design

A prospective quasi-experimental design was used, with baseline and follow-up data collection, pre- and post-establishment of the leadership hubs. Three intervention and three control districts were sampled in each country with the exception of South Africa where only intervention districts were included.^4^ One leadership hub was established in each intervention district. A process evaluation of the leadership hub intervention examined hub activities and self-rated changes in the capacity of hub members. Pre and post measures assessed district-wide changes in clinical practices, quality assurance, workplace policies and stigma by sampling health care institutions in intervention districts regardless of whether participants from those institutions had been involved as hub members.

### Multi-stage sampling

Intervention districts were purposively selected by country directors^5^ with consideration given to high HIV prevalence rates, proximity to the in-country research office, variations in geographic and socioeconomic conditions (e.g. urban versus rural), and the mix of health care institutions within the district. Control districts were selected on the basis of similarities in HIV rates and the distribution of health facilities. Because we aimed to improve district-wide HIV nursing care, eligibility for participation in pre and post measures was not limited only to the health facilities employing those hub members who were health care workers. All public government health care institutions within each study district were listed and categorised using WHO criteria for level of health facility [82]. Three levels of health facilities (WHO level 3, 4 and 5 institutions^6^) were eligible for inclusion. Level 3 facilities were health centres at the sub-district level (providing primarily health promotion and prevention services); level 4 facilities were district and sub-district hospitals; and level 5 facilities were referral hospitals at the provincial or national level. We excluded level 1 and 2 facilities (community health posts and dispensaries), and we excluded facilities that were officially designated as level 3 health centres but due to staffing limitations were actually functioning as a level 1 or 2 facility.

We used stratified, multi-stage random sampling. See Table 4 describing the number of institutions and participants by WHO institution level at baseline and follow-up. We enrolled 16 health care institutions (as planned) in both the intervention and control districts of each country (with the exception of South Africa where there were no control districts). All national, provincial and district hospitals that met the WHO criteria were included in the sample. In Kenya, a level 5 hospital that served the three control districts was located in an adjacent province and was included in the control sample. In Uganda, two national hospitals in the same intervention district were selected. One of these national hospitals was designated an intervention site, while the other, a specialty hospital, was assigned to the control group as it also provided services to clientele in the adjacent control district. Four or five health centres, which met WHO criteria for staff mix and functions were randomly sampled in each district.^7^

**Table 4:** Number of institutions and participants by WHO institution level at baseline and follow-up

Country^a^	National or provincial hospitals (WHO level 5)				District or parish hospitals (WHO level 4)				Health Centres(WHO level 3)				Totals at baseline and follow-up				Programme totals	
	Baseline		Follow-up		Baseline		Follow-up		Baseline		Follow-up		Baseline		Follow-up			
	# of instit.	# of part.	# of instit.	# of part.	# of instit.	# of part.	# of instit.	# of part.	# of instit.	# of part.	# of instit.	# of part.	# of instit.	# of part.	# of instit.	# of part.	# of instit.	# of part.
Jamaica^b^	5	150	5	95	2	17	2	40	18	49	23	62	25	216	30	197	31	413
Kenya^c^	2	131	2	136	5	25	5	60	22	61	19	80	29	217	26	276	31	493
Uganda^c^	2	143	2	140	6	49	1	5	24	84	16	75	32	276	19	220	32	496
South Africa^d^	2	81	2	37	1	30	1	29	12	46	12	54	15	157	15	120	15	277
Total	11	505	11	408	14	121	9	134	76	240	70	271	101	866	90	813	109	1679

Organisation and management of health services in partner countries during the study period was centred on the district level. Level 1 and 2 facilities included community health posts and dispensaries. Level 3 facilities were health centres at the sub-district level (providing primarily health promotion and prevention services). Level 4 facilities referred to district and sub-district hospitals providing curative services. Level 5 facilities were referral hospitals at the provincial or national level. This study involved health facilities at levels 3–5

*instit*. institutions, *part*. participants

^a^The same institutions for all countries were sampled at baseline and follow-up when possible. Most institutions have data at both baseline and follow-up; however, some institutions only have data at either baseline or follow-up

^b^Increases in number from pre to post were due to increased availability of institutions at post-data collection, which were unavailable during pre-data collection

^c^Decreases in numbers from pre to post were due to institutional losses

^d^South Africa had no control districts

National, provincial and district hospitals were provided with a protocol for randomly sampling staff. All eligible staff in health centres were invited to participate. Eligibility criteria included registered or enrolled nurse, staff nurse or manager; employed in their health care setting for at least 3 months; and fluent in English.

We used the same approach to sample participants for follow-up data collection. While the same institutions were sampled at both data collection points, the identity of respondents was anonymous, and thus, we were unable to match responses to baseline and follow-up surveys for those who participated in both data collection periods. Of the institutions sampled, 82 of the 109 institutions with valid survey data, or 75.2 %, had at least one respondent participate at both data collection periods. Three institutions did not have valid data at either collection point.

### Pre and post measures

The same self-administered questionnaire was completed at baseline and follow-up.

Socio-demographic characteristics included sex, professional designation (enrolled nurse, registered nurse), highest education level (diploma or certificate, higher level of education), current work location within the institution (community, obstetrics and/or gynaecology, other) and frequency of contact with HIV patients (daily, less often than daily).

We adapted validated instruments [62, 72, 83] to measure clinical practices, quality assurance initiatives and workplace policies. Participants were asked to report their own clinical practices and those of their nursing co-workers (12 parallel items for each scale) using a five-point Likert scale. Response options were never (1), rarely (2), sometimes (3), most of the time (4) and always (5). Ten-item scales were used to assess both workplace quality assurance initiatives and workplace policies. Response options were yes (1), no (2) or unsure (3).

We assessed two dimensions of HIV stigma and discrimination [84]: stigmatising by nurses against people with HIV (10 items) and stigmatising by co-workers and the community against nurses who provide care to people with HIV (9 items). Responses were captured using a four-point scale: never (1), once or twice (2), several times (3) and most of the time (4).

#### Data collection

Country RAs were trained by the principal investigators (NE, DK, EK) and project manager (SR) through a 1-week intensive session. Research staff were introduced to the protocol and to PAR, taught how to conduct field sampling and assess eligibility of health care institutions and participants, instructed on how to obtain consent and trained to use all data collection measures [77]. Prior to follow-up data collection, an updated field guide was developed and refresher training held. Day-to-day supervision of RAs was provided by country directors.

#### Data analysis of pre and post measures

**Fig. 2:**
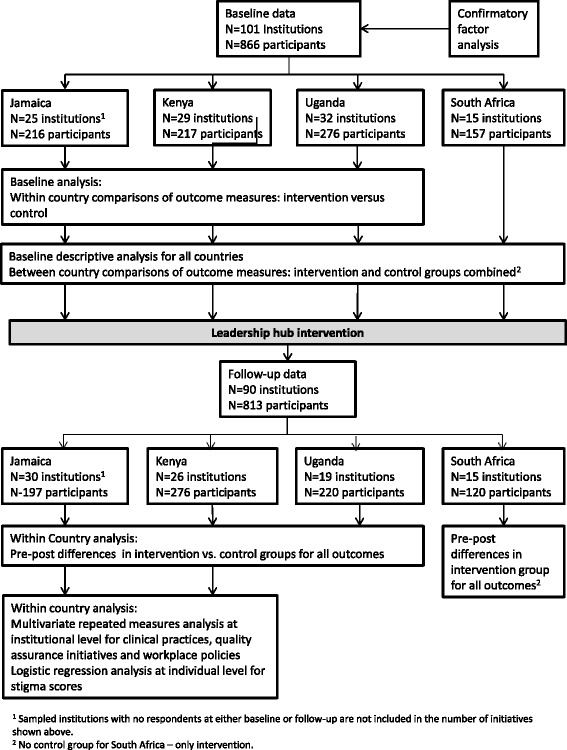
Data analysis process

Data were entered in an Excel database and cleaned by RAs at each country site. RAs in Jamaica and Kenya completed preliminary data analysis under the direction of the principal investigators. Final analysis using SPSS [85] and the R statistical programme [86] was undertaken with the assistance of a statistician who worked with the Canadian principal investigator (NE). Data analysis steps are shown in Fig. 2.

#### Descriptive and bivariate analysis

Descriptive analyses were used to initially examine data and to assess response patterns. A Pearson chi-square was used to compare socio-demographic characteristics of respondents in intervention and control districts at baseline and follow-up.

Several items were mistakenly excluded from one or more countries’ pre-intervention questionnaires. Consequently, we discarded one item from each of the clinical assessment scales and four items from both the work place policies and quality assurance scales. We then conducted confirmatory factor analysis on these four scales and determined that a one-factor solution was optimal for each. Confirmatory factor analysis on the stigma scales yielded the two-factor structure reported in the literature [84].

As recommended [84], we analysed scores on the stigma sub-scales in two ways, using the standardised mean score (ranging from 0 to 1) and a dichotomised score (never (0) versus one or more (1)). For clinical practice scores, we collapsed and recoded responses to the 11 items as never, rarely or sometimes (0), versus most of the time or always (1) and computed a standardised mean score (ranging from 0 to 1) for both scales. For workplace polices and quality assurance measures, we retained six items from each scale and compared yes (1) versus no or unsure (0) responses. If someone had more than one missing value, the entire response for that scale was deemed to be missing; otherwise, the missing item value was imputed using a means score imputation procedure.

Inter-country differences in baseline mean scores were assessed for all scales, with data from respondents in intervention and control groups combined, using ANOVA and post hoc testing (Tukey’s HSD and Scheffe’s tests). We used a paired *t* test to compare respondents’ scores on the two stigma sub-scales and on quality assurance and workplace policies.

Within-country, pre versus post differences in outcomes between intervention and control groups for Jamaica, Kenya and Uganda, were assessed using the Mantel Haenszel chi-square (for dichotomised stigma scores) and independent *t* tests on the pre-post differences in mean scores between intervention and control groups (for all other measures). For South Africa, which had no control group, pre-post differences were compared using a Pearson chi-square and independent *t* test.

#### Multivariate analysis

Multivariate analysis was completed for Kenya and Jamaica. Uganda was excluded due to a large amount of missing data for district hospitals at follow-up. South Africa was excluded as there was no control group.

Stigma measures were analysed at the individual level, using a fixed effects logistic model. We analysed other outcomes using a repeated measures model and aggregated the data by institutions as this better met the assumptions of normality. This yielded one response for each institution and period. Institutions with surveys from only one period were discarded. Precision weights based on the sample size were used to account for varying numbers of surveys from different institutions and periods.

Common parameters for all models were (a) WHO institution (national/provincial, district and health centre); (b) intervention group (control and intervention, ignoring period); (c) period (pre (baseline) and post (follow-up)); and (d) intervention period, which had one non-zero value for any additional change in the control districts in the post period over what would occur if intervention and period operated independently.

### Process evaluation of leadership hubs

#### Data collection

The characteristics of hub members and composition of hubs were documented by RAs in annual progress reports. The number and content of hub meetings and training sessions was captured from bi-monthly structured reports completed by RAs. Near the end of the project, each hub member was asked to rate their capacity (pre and post hub experience) on seven skill dimensions using a 10-point Likert scale (1 = low capacity, 10 = high capacity).

#### Data analysis

Turnover rates in hub membership were estimated using standard labour force methods [87] to assess period turnover: *L*/[(*N*(*i*) + *N*(*f*))/*2*], where *L* = number of hub members who left during the period; *N*(*i*) = number of hub members at the beginning of the period; and *N*(*f*) = number of hub members at the end of the period. Membership counts were determined for the beginning and end of each period using information in bi-monthly hub reports. At follow-up, hub members were categorised as active if they were listed as contributors to the final evaluation project reports.

Average mean scores for each capacity dimension were calculated. Pre-post differences were compared using a paired *t* test.

## Results

### Leadership hubs

At follow-up, leadership hubs averaged 8.4 members per hub; a total of 167 members participated in the hubs over the course of the intervention period. Over half (58.9 %) of hub members were direct care nurses and nurse managers; 11 % were researchers; 31.5 % were decision-makers; and 21.9 % were community representatives. The majority of hub members came from non-sampled health institutions (49.1 %) or from other workplaces or the community (22.2 %). The remainder (28.7 %) came from the district health institutions sampled for pre and post measures. Over one third (35.9 %) of sampled institutions in intervention districts had hub member representation; Jamaica had the largest portion of hub members (57.1 %) from sampled institutions. In six of the hubs, members included one person self-disclosed as living with HIV. Table 5 describes the membership characteristics of leadership hubs by country.

**Table 5:** Membership and characteristics of leadership hubs, 2008–2012

Hub characteristics and functions	Jamaica	Kenya	Uganda	South Africa	All countries
Average number of years hubs in each country that were operational during programme	3.9	4.0	4.1	3.3	3.8
Number of hubs operational at follow-up	3/3	3/3	3/3	2/3	11/12
Total number of hub members over hub lifespan	28	58	33	48	167
Average number of members per hub (*n* = total number of hub members from all hubs in country)					
At baseline	7.0 (*n* = 21)	13.7 (*n* = 41)	7.0 (*n* = 21)	9.0 (*n* = 27)	9.1 (*n* = 110)
At follow-up^d^	5.3 (*n* = 16)	9.7 (*n* = 29)	6.7 (*n* = 20)	13.5 (*n* = 27)^a^	8.4 (*n* = 92)^a^
Percentage of hub members at follow-up actively participating in hub activities	100 %	69.0 %	95.0 %	66.7 %	79.3 %
Composition of hub members actively participating in hub activities at follow-up^b^					
Nurses	12 (75.0 %)	17 (85.0 %)	7 (36.8 %)	7 (38.9 %)	43 (58.9 %)
Decision-makers	0 (0.0 %)	10 (50.0 %)	7 (36.8 %)	6 (33.3 %)	23 (31.5 %)
Researchers	3 (18.8 %)	0 (0.0 %)	5 (26.3 %)	0 (0.0 %)	8 (11.0 %)
Community representatives	1 (6.3 %)	3 (15.0 %)	6 (31.6 %)	4 (22.2 %)	14 (19.2 %)
Leadership hub turnover rate^c^					
2008–2009^d^	0.0 %	27.3 %	8.0 %	9.4 %	13.5 %
2010	34.1 %	2.8 %	10.9 %	10.8 %	12.4 %
2011	0.0 %	24.6 %	0.0 %	6.3 %	10.0 %
2012^d,e^	27.0 %	0.0 %	0.0 %	0.0 %	5.0 %
Average over hub lifespan 95 % confidence interval	15.3 ± 15.2 %	13.7 ± 12.1 %	4.7 ± 4.7 %	6.6 ± 4.1 %	10.2 ± 3.2 %
Percentage of hub members at follow-up that were in the hub for its total duration	81.3 % (13/16)	65.5 % (19/29)	65.0 % (13/20)	77.8 % (21/27)	71.7 % (66/92)
Number of sampled intervention institutions with hub member representatives engaged in the hub evaluation projects	4	8	2	3	17
Percentage of hub members over hub lifespan from					
Sampled institutions	57.1 % (16/28)	19.0 % (11/58)	24.2 % (8/33)	27.1 % (13/48)	28.7 % (48/167)
Non-sampled institutions	39.3 % (11/28)	56.9 % (33/58)	51.5 % (17/33)	43.8 % (21/48)	49.1 % (82/167)
Other workplaces or community	3.6 % (1/28)	24.1 % (14/58)	24.2 % (8/33)	29.2 % (14/48)	22.2 % (37/167)
Percentage of sampled intervention institutions with hub members representation during the hub lifespan					
National or provincial hospitals	100 % (3/3)	100 % (1/1)	100 % (1/1)	50 % (1/2)	85.7 % (6/7)
District or parish hospitals	100 % (1/1)	66.7 % (2/3)	33.3 % (1/3)	0 % (0/1)	50.0 % (4/8)
Health centres	0 % (0/13)	50.0 % (6/12)	0.0 % (0/12)	58.3 % (7/12)	26.5 % (13/49)
Average for all institutions	23.5 % (4/17)	56.3 % (9/16)	12.5 % (2/16)	53.3 % (8/15)	35.9 % (23/64)
Range and average number of hub members per intervention institution during the hub lifespan	Range 0–9	Range 0–2	Range 0–5	Range 0–4	Range 0–9
	Average 0.9	Average 0.7	Average 0.5	Average 0.9	Average 0.8
Range and average number of distinct workplaces represented by hub members in each hub during hub lifespan	Range 2–5	Range 8–11	Range 3–7	Range 8–15	Range 2–15
	Average 3.3	Average 9.7	Average 4.7	Average 11.7	Average 7.4
Number of hub meetings per country, 2008–2012	30	39	25	36	130

^a^One of the South African hubs ceased operations at the end of 2011 so was not included in follow-up measures

^b^Categories are not mutually exclusive; some hub members were listed in more than one category

^c^Leadership hub turnover is calculated as *L*/[(*N*(*i*) + *N*(*f*))/2], where *L* = number of hub members who left during the period; *N*(*i*) = number of hub members at the beginning of the period; and *N*(*f*) = number of hub members at the end of the period. (Bureau of Labor Statistics, n.d.)

^d^Since hubs were established in either 2008 or 2009 (depending on the hub and country), turnover data for 2008 and 2009 were collapsed

^e^The final period was 6 months (January–June 2012)

### Sample for pre and post measures

A total of 101 and 90 health care institutions participated at baseline and follow-up, respectively (response rates of 90.2 and 80.4 %). There were 866 survey respondents at baseline and 813 at follow-up. A minority of follow-up participants (ranging from 12.4 % in Jamaica to 25 % in South Africa) indicated that they had also completed the baseline interview. All study districts had level 3 health centres (30.4 % of the sample) but some districts also had a level 5 provincial/national hospital and/or a level 4 district hospital (54.5 and 15.2 % of participants, respectively). In Uganda at follow-up, there were no participants from district hospitals (level 4 institutions) in the control group and only five in the intervention group (see Table 4).

Baseline respondents were predominantly female, and 77.2 % were registered nurses. The majority of respondents (71.7 %) had at least daily contact with HIV patients. Participants had worked in their current work setting an average of 9.75 years; 49.3 % of the participants were in a staff nurse position and 50.7 % were managers. Kenya had the largest percentage of male respondents. South Africa had the largest proportion of registered nurses, while Jamaica had the largest proportion of enrolled nurses. The proportion of registered nurses was highest in health centres in both Jamaica and Kenya. Jamaican respondents reported significantly less daily contact with HIV patients (35.2 %) compared with other countries (over 80 %) (see Table 6 for socio-demographic characteristics of all health workers by country and by data collection period).

**Table 6:** Socio-demographic characteristics of participants on clinical practices, quality assurance, workplace policies and stigma scales. Data included is from all participants who completed pre or post questionnaires for the listed measures. For Jamaica, Kenya and Uganda, the following within-country comparisons were calculated: intervention (pre versus post); control (pre versus post); and pre-intervention versus pre-control versus post-intervention versus post-control. For South Africa, a pre versus post comparison was included

	Jamaica							Kenya							Uganda							South Africa		
	Intervention			Control			Int vs. con^a^	Intervention			Control			Int vs. con^a^	Intervention			Control			Int vs. con^a^	Intervention		
Socio-demographic characteristic	Pre *n* (%)	Post *n* (%)	*χ* ^2^ sig	Pre *n* (%)	Post *n* (%)	*χ* ^2^ sig	*χ* ^2^ sig	Pre *n* (%)	Post *n* (%)	*χ* ^2^ sig	Pre *n* (%)	Post *n* (%)	*χ* ^2^ sig	*χ* ^2^ sig	Pre *n* (%)	Post *n* (%)	*χ* ^2^ sig	Pre *n* (%)	Post *n* (%)	*χ* ^2^ sig	*χ* ^2^ sig	Pre *n* (%)	Post *n* (%)	*χ* ^2^ sig
Total	121	93	–	95	104	–	–	100	161	–	117	115	–	–	135	103	–	141	117	–	–	157	120	–
Sex																								
Female	119 (98.3)	92 (98.9)	0.722	94 (98.9)	104 (100)	0.294	0.742	79 (79.0)	137 (85.1)	0.442	99 (84.6)	89 (77.4)	0.088	0.577	131 (97.0)	98 (95.1)	0.686	128 (90.8)	103 (88.0)	0.745	0.763	148 (94.3)	108 (90.0)	0.265
Male	2 (1.7)	1 (1.1)		1 (1.1)	0 (0)			18 (18.0)	24 (14.9)		16 (13.7)	26 (22.6)			4 (3.0)	4 (3.9)		13 (9.2)	12 (10.3)			9 (5.7)	11 (9.2)	
Missing^b^	0 (0)	0 (0)	N/A	0 (0)	0 (0)	N/A	N/A	3 (3.0)	0 (0)	N/A	2 (1.7)	0 (0)	N/A	N/A	0 (0)	1 (1.0)	N/A	0 (0)	2 (1.7)	N/A	N/A	0 (0)	1 (0.8)	N/A
Level of health facility																								
National/provincial	84 (69.4)	47 (50.5)	0.008	66 (69.5)	48 (42.1)	0.002	N/A	49 (49.0)	74 (46.0)	0.270	82 (70.1)	62 (53.9)	0.017	N/A	69 (51.1)	77 (74.8)	0.000	74 (52.5)	63 (53.8)	0.000	N/A	81 (51.6)	37 (30.8)	0.002
District/parish	10 (8.3)	19 (20.4)		7 (7.4)	21 (20.2)			16 (16.0)	39 (24.2)		9 (7.7)	21 (18.3)			24 (17.8)	5 (4.9)		25 (17.7)	0 (0)			30 (19.1)	29 (24.2)	
Health centre	27 (22.3)	27 (29.1)		22 (23.1)	35 (33.7)			35 (35.0)	48 (29.8)		26 (22.2)	32 (27.8)			42 (31.1)	21 (20.4)		42 (29.8)	54 (46.2)			46 (29.3)	54 (45.0)	
Professional designation																								
Enrolled nurse/midwife	33 (27.3)	17 (18.3)	0.157	21 (22.1)	14 (13.5)	0.123	0.051	–	39 (24.2)	N/A	44 (37.6)	31 (27.4)	0.000	N/A	–	11 (10.7)	N/A	–	49 (41.9)	N/A	N/A	0 (0)	9 (7.6)	0.004
Registered nurse/midwife	88 (72.7)	73 (78.5)		73 (76.8)	87 (83.7)			––	119 (74.0)		36 (30.8)	82 (72.6)			–	87 (84.5)		–	50 (42.7)			102 (65.0)	109 (90.8)	
Missing^b^	0 (0)	3 (3.2)	N/A	1 (1.1)	3 (2.8)	N/A	N/A	100 (100)	3 (1.8)	N/A	37 (31.6)	2 (1.7)	N/A	N/A	135 (100)	5 (4.9)	N/A	141 (100)	18 (15.4)	N/A	N/A	55 (35.0)	2 (1.6)	N/A
Highest education level																								
Diploma/certificate	87 (72.0)	44 (47.3)	0.001	65 (68.4)	65 (62.5)	0.530	0.005	53 (53.0)	137 (85.1)	0.009	82 (70.1)	84 (73.1)	0.042	0.792	89 (65.9)	92 (89.3)	0.291	69 (48.9)	101 (86.3)	0.625	0.350	81 (51.6)	84 (70.0)	0.029
Higher level of education	33 (27.2)	45 (48.4)		28 (29.5)	34 (32.7)			22 (22.0)	24 (14.9)		13 (11.1)	28 (24.3)			14 (10.4)	9 (8.7)		11 (7.8)	13 (11.1)			57 (36.3)	33 (27.5)	
Missing^b^	1 (0.8)	4 (4.3)	N/A	2 (2.1)	5 (4.8)	N/A	N/A	25 (25.0)	0 (0)	N/A	22 (18.8)	3 (2.6)	N/A	N/A	32 (23.7)	2 (1.9)	N/A	61 (43.3)	3 (2.6)	N/A	N/A	19 (12.1)	3 (2.5)	N/A
Current work location within the institution																								
Community	29 (24.0)	25 (26.9)	0.083	18 (18.9)	27 (26.0)	0.027	N/A	7 (7.0)	7 (4.3)	0.154	13 (11.0)	5 (4.3)	0.003	N/A	3 (2.2)	8 (7.8)	0.002	15 (10.6)	6 (5.1)	0.197	N/A	49 (31.2)	38 (31.7)	0.988
Obstetrics/gynaecology	6 (5.0)	12 (12.9)		4 (4.2)	14 (13.5)			7 (7.0)	23 (14.3)		20 (17.1)	7 (6.1)			36 (26.7)	11 (10.7)		5 (3.5)	7 (6.0)			18 (11.5)	14 (11.7)	
Other^c^	84 (69.3)	55 (59.1)		70 (73.7)	62 (59.6)			84 (84.0)	130 (80.7)		83 (71.0)	103 (89.6)			96 (71.1)	83 (80.6)		118 (83.7)	101 (86.3)			87 (55.4)	65 (54.2)	
Missing^b^	2 (1.7)	1 (1.1)	N/A	3 (3.2)	1 (1.0)	N/A	N/A	2 (2.0)	1 (0.7)	N/A	1 (0.9)	0 (0)	N/A	N/A	0 (0)	1 (0.9)	N/A	3 (2.1)	3 (2.6)	N/A	N/A	3 (1.9)	3 (2.5)	N/A
Contact with patients/clients with HIV or AIDS																								
Daily	51 (42.1)	37 (39.8)	0.633	23 (24.2)	33 (31.7)	0.206	0.703	91 (91.0)	146 (90.7)	0.561	94 (80.3)	98 (85.2)	0.674	0.588	107 (79.3)	68 (66.0)	0.022	114 (80.9)	75 (64.1)	0.002	0.000	136 (86.6)	109 (90.8)	0.556
Less often than daily^d^	65 (53.7)	54 (58.1)		71 (74.7)	68 (65.4)			9 (9.0)	11 (6.8)		19 (16.2)	17 (14.8)			27 (20.0)	34 (33.0)		25 (17.7)	40 (34.2)			16 (10.2)	10 (8.3)	
Missing^b^	5 (4.1)	2 (2.2)	N/A	1 (1.1)	3 (2.9)	N/A	N/A	0 (0)	4 (2.5)	N/A	4 (3.4)	0 (0)	N/A	N/A	1 (0.7)	1 (1.0)	N/A	2 (1.4)	2 (1.7)	N/A	N/A	5 (3.2)	1 (0.8)	N/A

*vs*. versus

^a^Int vs. con comparison is a Mantel Haenszel chi-square statistics; unable to perform for South Africa due to no control group; unable to perform for health facility level and work location due to more than two distinct variables

^b^Missing data was not included in calculations of significance levels

^c^Includes accident/emergency/casualty, medical/surgical, outpatient/ambulatory car, operating theatre, intensive care unit or other as indicated by participants

^d^Weekly, monthly, several times per year or never

In all countries, there were significant differences in the composition of the sample coming from different WHO institutional levels between baseline and follow-up, and in the three countries with control groups, between intervention and control districts. WHO institution level was associated with significant differences in baseline scores for most outcomes (clinical practices (self and peers), quality assurance and workplace policies were assessed): Jamaica (4/4), Kenya (2/4), South Africa (4/4) and Uganda (3/4). However, the direction of these differences varied. In Jamaica, Kenya and South Africa, health centres had the highest (best) scores while, in Uganda, health centres had the lowest (worst) scores (see Table 7).

**Table 7:** Outcome measures for the clinical practice—self, clinical practice—peers, quality assurance and workplace policies scales. For Jamaica, Kenya and Uganda, the following within-country comparisons were calculated: pre (intervention versus control); and pre-intervention versus pre-control versus post-intervention versus post-control. For South Africa, a pre versus post comparison was included

Scale		Jamaica					Kenya					Uganda					South Africa	
		Pre (int. vs. con.)	Intervention		Control		Pre (int. vs con.)	Intervention		Control		Pre (int. vs con.)	Intervention		Control		Intervention	
			Pre	Post	Pre	Post		Pre	Post	Pre	Post		Pre	Post	Pre	Post	Pre	Post
Total		216	121	93	95	104	217	100	161	117	115	276	135	103	141	117	157	120
Clinical practices—self	Number	205	115	89	90	97	206	97	160	109	113	270	135	99	135	117	155	117
	Missing	11	6	4	5	7	11	3	1	8	2	6	0	4	6	0	2	3
	*μ*(SD)	0.46 (0.26)	0.44 (0.24)	0.42 (0.25)	0.50 (0.28)	0.47 (0.25)	0.71 (0.24)	0.70 (0.25)	0.76 (0.25)	0.71 (0.24)	0.73 (0.24)	0.56 (0.24)	0.59 (0.24)	0.64 (0.28)	0.53 (0.24)	0.56 (0.28)	0.67 (0.29)	0.78 (0.24)
	95 % CI	0.43–0.50	0.40–0.48	0.37–0.48	0.44–0.56	0.42–0.52	0.67–0.74	0.65–0.75	0.72–0.80	0.67–0.76	0.69–0.77	0.53–0.58	0.55–0.63	0.58–0.69	0.48–0.57	0.51–0.61	0.62–0.72	0.73–0.82
	*p* value^a^	0.121	0.8831				0.809	0.3988				0.038	0.7663				0.002	
Clinical practices—peers	Number	197	112	83	85	95	209	98	156	111	111	273	135	99	138	117	153	117
	Missing	19	9	10	10	9	8	2	5	6	4	3	0	4	3	0	4	3
	*μ*(SD)	0.54 (0.29)	0.51 (0.29)	0.48 (0.28)	0.58 (0.28)	0.53 (0.30)	0.73 (0.25)	0.76 (0.24)	0.81 (0.25)	0.71 (0.25)	0.76 (0.25)	0.60 (0.24)	0.63 (0.24)	0.63 (0.30)	0.57 (0.24)	0.65 (0.29)	0.72 (0.30)	0.79 (0.27)
	95 % CI	0.50–0.58	0.46–0.57	0.42–0.54	0.52–0.65	0.45–0.58	0.70–0.77	0.71–0.81	0.77–0.85	0.67–0.76	0.71–0.81	0.57–0.63	0.59–0.67	0.57–0.69	0.53–0.61	0.59–0.70	0.68–0.77	0.74–0.83
	*p* value^a^	0.086	0.5527				0.229	0.7896				0.036	0.1199				0.098	
Quality assurance	Number	210	116	92	94	101	210	99	161	111	113	268	133	102	135	116	154	118
	Missing	6	5	1	1	3	7	1	0	6	2	8	2	1	6	1	3	2
	*μ*(SD)	0.71 (0.28)	0.67 (0.29)	0.69 (0.30)	0.76 (0.25)	0.67 (0.30)	0.72 (0.30)	0.77 (0.29)	0.82 (0.21)	0.67 (0.31)	0.81 (0.24)	0.49 (0.29)	0.52 (0.31)	0.56 (0.30)	0.45 (0.27)	0.74 (0.26)	0.72 (0.31)	0.84 (0.22)
	95 % CI	0.67–0.75	0.61–0.72	0.63–0.75	0.70–0.80	0.61–0.73	0.68–0.76	0.72–0.83	0.78–0.85	0.62–0.73	0.77–0.86	0.45–0.52	0.47–0.57	0.50–0.62	0.41–0.50	0.70–0.79	0.67–0.77	0.80–0.88
	*p* value^a^	0.023	0.0262				0.016	0.0401				0.069	0.0001				0.001	
Workplace policies	Number	208	115	92	93	98	210	100	159	110	114	271	133	101	138	115	153	119
	Missing	8	6	1	2	6	7	0	2	7	1	5	2	2	3	2	4	1
	*μ*(SD)	0.80 (20)	0.78 (0.22)	0.81 (0.21)	0.84 (0.19)	0.77 (0.21)	0.85 (0.20)	0.89 (0.16)	0.86 (0.17)	0.82 (0.27)	0.87 (0.18)	0.56 (0.32)	0.65 (0.32)	0.62 (0.31)	0.47 (0.31)	0.76 (0.26)	0.82 (0.22)	0.87 (0.17)
	95 % CI	0.78–0.83	0.74–0.82	0.77–0.85	0.80–0.88	0.73–0.81	0.82–0.88	0.86–0.93	0.83–0.88	0.77–0.86	0.84–0.91	0.52–0.59	0.59–0.70	0.56–0.68	0.42–0.52	0.71–0.81	0.79–0.85	0.84–0.90
	*p* value^a^	0.025	0.0121				0.006	0.540				0.000	0.0001				0.031	

*vs*. versus

^a^The *p* values displayed in the “Pre” columns are a comparison of mean scores between intervention and control districts at baseline. These were calculated using an unpaired *t* test. The other *p* values, which are displayed under the intervention/control columns, are a comparison of mean differences in the pre-post scores for intervention versus control groups. *p* values for (Pre-Int vs. Pre-Con vs. Post-Int vs. Post-Con) for Kenya, Jamaica and Uganda were calculated using an online *t* test tool; *p* values for South Africa were calculated using an unpaired *t* test

### Inter-country comparisons at baseline

At baseline, Jamaica and Uganda had significantly lower (worse) clinical practice scores than either Kenya or South Africa (assessment of self and peers; *F* = 37.1, *p* < 0.0001; *F* = 25.1, *p* < 0.0001, respectively), while Uganda had significantly lower scores on workplace policies and quality assurance than other countries (*F* = 37.4, *p* < 0.00001; and *F* = 74.8, *p* < 0.00001, respectively). All four countries had significantly worse scores for quality assurance initiatives than workplace policies (*p* < 0.0001).

A comparison of scale items revealed four main practice gaps across all countries. These were as follows: assessing the knowledge of family members to prevent HIV transmission, clients’ comfort in disclosing their HIV status to family members, family health needs related to HIV care; and referring family members for voluntary counselling and testing. A similar pattern was observed for related items assessing quality assurance initiatives and workplace policies.

The highest (best) score on the clinical practice scale was for the item “consistently using universal precautions to prevent HIV transmission with clients” (>90 % for all countries). However, quality assurance initiatives to monitor either adherence to universal precautions or the occurrence of occupational exposure to HIV were more variable, ranging within country districts from 47.2 to 80.7 % and 56 to 81 %, respectively.

At baseline, stigma scores on both sub-scales were significantly different across countries. Post hoc analysis indicated that Uganda and Kenya had worse scores on the nurses’ stigmatising scale than South Africa. On the nurses being stigmatised scale, Jamaica had significantly better scores, while Uganda had significantly worse scores than comparator countries. Nurses were significantly more likely to report being stigmatised than stigmatising patients (*p* < 0.0001) in all countries except Jamaica, where the reverse relationship was found (*p* = 0.002) (see Table 8).

**Table 8:** Mean scores and occurrences for the two stigma sub-scales. For Jamaica, Kenya and Uganda, the following within-country comparisons were calculated: pre-intervention versus pre-control versus post-intervention versus post-control. For South Africa, a pre versus post comparison was calculated

		Jamaica					Kenya					Uganda					South Africa		
		Intervention		Control		Int. vs. con *p* value	Intervention		Control		Int. vs. con *p* value	Intervention		Control		Int. vs. con *p* value	Intervention		Pre vs. post *p* value
		Pre	Post	Pre	Post		Pre	Post	Pre	Post		Pre	Post	Pre	Post		Pre	Post	
Total # of participants		121	93	95	104		100	161	117	115		135	103	141	117		157	120	
Nurses stigmatising patients																			
Mean score (SD)^a^		0.293 (0.40)	0.180 (0.46)	0.220 (0.43)	0.163 (0.40)	0.570	0.271 (0.35)	0.199 (0.35)	0.399 (0.49)	0.235 (0.34)	0.201	0.242 (0.41)	0.175 (0.36)	0.438 (0.68)	0.224 (0.34)	0.089	0.202 (0.40)	0.145 (0.24)	0.003
Count of instances *n*(%)^b^	Never	41 (33.9)	58 (62.4)	52 (54.7)	67 (64.4)	0.002	35 (35.0)	83 (51.6)	40 (34.2)	55 (47.8)	0.006	64 (47.4)	58 (56.3)	66 (46.8)	59 (50.4)	0.279	91 (58.0)	59 (49.2)	0.182
	One or more	60 (49.6)	32 (34.4)	32 (33.7)	28 (26.9)		58 (58.0)	75 (46.6)	69 (59.0)	59 (51.3)		70 (51.9)	44 (42.7)	70 (49.6)	58 (49.6)		62 (39.5)	56 (46.7)	
	Missing	20 (16.5)	3 (3.2)	11 (1.6)	9 (8.7)		7 (7.0)	3 (1.9)	8 (6.8)	1 (0.9)		1 (0.7)	1 (1.0)	5 (3.5)	0 (0)		4 (2.5)	5 (4.2)	
Nurses being stigmatised																			
Mean score (SD)^a^		0.195 (0.32)	0.151 (0.36)	0.113 (0.26)	0.138 (0.30)	0.267	0.599 (0.57)	0.567 (0.68)	0.713 (0.65)	0.648 (0.70)	0.737	0.841 (0.65)	0.653 (0.60)	1.331 (0.76)	0.750 (0.642)	0.002	0.673 (0.80)	0.565 (0.76)	0.502
Count of instances *n*(%)^b^	Never	64 (52.9)	61 (65.6)	60 (63.2)	68 (65.4)	0.148	23 (23.0)	60 (37.3)	18 (15.4)	31 (27.0)	0.004	15 (11.1)	18 (17.5)	9 (6.4)	18 (15.4)	0.014	48 (30.6)	45 (37.5)	0.192
	One or more	52 (43.0)	28 (30.1)	30 (31.6)	32 (30.8)		73 (73.0)	99 (61.5)	92 (78.6)	84 (73.0)		118 (87.4)	83 (80.6)	127 (90.1)	98 (83.8)		106 (67.5)	71 (59.2)	
	Missing	5 (4.1)	4 (4.3)	5 (5.3)	4 (3.8)		4 (4.0)	2 (1.2)	7 (6.0)	0 (0)		2 (1.5)	2 (1.9)	5 (3.5)	1 (0.9)		3 (1.9)	4 (3.3)	

*vs*. versus

^a^Data used to calculate mean scores were collected via a Likert scale. Significance figures for mean scores were calculated as intervention significance versus control significance using an unpaired *t* test. A pre versus post was used for South Africa due to no control group. Missing data were excluded

^b^Data used to calculate the count of instances was collected on a nominal scare. Significance figures for count of instances calculated using a Mantel Haenszel chi-square for Kenya, Jamaica and Uganda; a Pearson chi-square was used for South Africa. Missing data were excluded from calculations

### Pre-post differences

South Africa was the only country with a significant improvement in clinical practices from baseline to follow-up (*p* = 0.0002) (see Table 7). There were statistically significant improvements in quality assurance and workplace policies for Jamaica and South Africa (*p* = 0.026 and *p* = 0.001 for quality assurance and *p* = 0.025 and *p* = 0.031 for workplace policies, respectively). In Jamaica, significance levels reflected slight improvements in the intervention group in combination with deteriorations in the control group. Both Kenya and Uganda experienced a significant deterioration in quality assurance scores in intervention relative to control districts from pre to post periods. In Uganda, the same significant decline was seen for workplace policies.

Using the dichotomised score for stigma, both Jamaican and Kenyan participants in intervention institutions reported a significant decrease in pre to post instances of stigmatising HIV patients compared to those in control institutions (see Table 8). Kenyan and South African nurses reported a decline in instances of being stigmatised between pre and post periods. However, in Uganda, the control group reported a significantly larger pre-post reduction in being stigmatised than the intervention group. Overall, there was a downward trend in reports of nurses stigmatising patients in both intervention and control groups in Uganda and Kenya and in intervention groups in South Africa and Jamaica.

### Multivariate analysis

There were 24 Jamaican institutions and 24 Kenyan institutions with data for both periods. The intervention effect was not statistically significant for any of the measures. Uganda was not included in this analysis due to the large amount of missing data at follow-up resulting from an administrative error, specifically, data not being collected from provincial/district hospitals (level 4). South Africa did not have a control group due to budgetary constraints and was therefore also excluded from this analysis.

### Process evaluation

#### Hub sustainability and turnover rates

Twelve leadership hubs were set up in intervention districts across four countries. Eleven of 12 hubs remained active through to the end of the project, an average span of 3.8 years (one hub in South Africa became inactive before the project ended, after 2.8 years). The average turnover rate for hub members across all countries was 10.1 %. In the last 18 months, Kenya and Jamaica experienced the highest turnover rates. Primary reasons for turnover were a job transfer followed by being too busy or losing interest. The proportion of hub members reported as active in the final year of the programme ranged from 42.2 to 88.2 %. The proportion of hub members at follow-up who had been members since baseline ranged from 65.0 % (Uganda) to 81.3 % (Jamaica).

#### Hub training

Training progressed at different rates across countries with some locally driven variations in the sequence and selection of workshop topics, as described earlier. Not all hub members participated in all training workshops, primarily due to turnover. Each workshop was presented once, so members who were unavailable on the workshop day or who joined the hub at a later date missed that formal training session. During their second year of operation, all hubs developed district-focused health action plans targeting gaps identified in the baseline findings. They were encouraged to develop plans that included all institutions in the district, not just those that had been sampled for data collection. However, some hubs decided to focus their action on a particular tier of health facilities within their district (e.g. health centres). Hub members sometimes requested additional analyses from the research team to inform their action plans. These plans focused on issues including improving infection control; providing support activities for HIV-positive nurses; sensitising nurses to HIV-related stigma; and increasing nurses’ awareness of existing HIV workplace policies. Some plans included activities aimed at health issues other than HIV. Despite a common template that was used to guide hubs in developing plans, we observed unevenness in the specificity and level of activity proposed across action plans.

In the final year, 11 hubs completed a total of nine evaluation projects, all with a quality assurance focus (see Table 9). Hubs worked either singly or with other hubs in their country as they completed data collection, analysis and report-writing. The dissemination of findings from these projects took place in the last few months of the programme and in some cases continued after the programme ended. The proportion of intervention institutions that participated in the evaluation projects ranged from 35.3 % (Jamaica) to 88.2 % (Kenya).

**Table 9:** Leadership hub evaluation projects

Country	Leadership hub	Amount of grant (CAD)	Timeline	Project	Main findings and conclusions
Jamaica	St. Thomas Hub	$847	May 2011–November 2011	Observation of infection control procedures and interviews with staff to assess the implementation process of Jamaica’s National Infection Control Policy in parish health centres	Infection control committees were vibrant and active; infection control nurses deliberately assigned; inadequate supplies to maintain policy standards; insufficient allocation of coordinators; lack of coordinated approach to training
	St. Catherine Hub	$866	May 2011–February 2012	Analysis of hospital records and personnel training to assess the implementation of the voluntary counselling and testing (VCT) component of the Jamaican National HIV/AIDS policy in a parish hospital maternity unit	Extensive gaps in implementation and monitoring of VCT policy; no inclusion of VCT in orientation of new staff; low levels of VCT training; no committee to ensure VCT implementation
	Kingston and St. Andrew Hub	$993	May 2011–November 2011	Analysis of cases of occupational exposure to HIV (collected through required reporting and hospital injury records) to assess the adherence to post-exposure prophylaxis protocol in parish hospitals	Protocols for occupational injuries followed in some cases but not all; administration of post-exposure prophylaxis medication followed more closely than administrative aspects
Kenya	Suba Hub	$834	July 2011–March 2012	Surveys and interviews with hospital staff and clients to assess the impact of Kenya’s Service Charter on Health Sector Service Provision within district hospitals	Staff are knowledgeable and are partially implementing the service charter; there are a number of challenges preventing full implementation: human resources, finances, equipment and supplies; client satisfaction is satisfactory (60 %)
	Nyando Hub and Kisumu Hub	$1490	July 2011–March 2012	Structured questionnaires (with frontline nurses and nurse managers), and semi-structured exit interviews (with clients) to assess the implementation of Kenya’s National Reproductive Health Policy in Promotion of Safe Motherhood within country health facilities	HIV/AIDS components of the reproductive health policy are being implemented, with some exceptions; client satisfaction was above average, but there was room for improvement in some areas
Uganda	Kampala Hub	$773	May 2011–January 2012	Key informant interviews and focus group discussions to determine effective dissemination strategies to involve nurses and midwives in HIV workplace policies in district health centres	Most nurses and midwives are not well conversant with HIV workplace policies; health facilities that have policies do not have them in written documents; there is a strong need to improve dissemination strategies of HIV workplace policies to nurses and midwives
	Jinja Hub	$713	November 2011–February 2012	Structured interviews with nurses for the identification of nurse-designed best practices for addressing HIV stigma among nurses in district health centres	Top-down efforts to reduce stigma have failed to yield significant results; leaders at various levels need to be involved in stigma reduction
	Luwero Hub	$794	June 2011–January 2012	Questionnaires and focus group discussions with nurses to assess health workers’ knowledge, attitudes and practices towards implementation of universal safety precautions (USP) policy in district health facilities	High (93 %) knowledge of USP policy, but low (10 %) use of guidelines among nurses; resources needed for implementation of policies are often lacking; need for both dissemination of policy guidelines and supplies to implement
South Africa	Ngaka Modiri Molema Hub and Kenneth Kaunda Hub	$1855	June 2011–February 2012	Literature review and concept analysis on anti-retroviral therapy (ART) adherence and follow-up to develop a checklist tool for ART follow-up evaluation	Literature review showed that not all policies in place in institutions, and policies often not implemented, well-known, or used; no policy enforcement at the institutional level; current policies focus on accessibility and management of medications only

Leadership hub evaluation projects were funded by the study (“Strengthening Nurses’ Capacity in HIV Policy Development in Sub-Saharan Africa and the Caribbean”), which itself was funded by the Global Health Research Initiative (GHRI), a collaborative research funding partnership of the Canadian Institutes of Health Research, the Canadian International Development Agency, Health Canada, the International Development Research Centre, and the Public Health Agency of Canada [grant number 103460-042]

By the end of the programme, leadership hub members in each country reported statistically significant improvements in their self-rated capacity to identify and act on gaps in clinical care and health system issues for each of the seven capacity dimensions assessed (see Table 10). Pre-post change scores ranged from 3.59 to 4.59 (on a 10-point scale) for the eight hubs included in this analysis. The largest change score was reported for the competency “initiating and undertaking an evaluation project”. Data from two hubs were excluded from significance testing, as only aggregated data, rather than individual data, were provided by the RA. Competency scores from these two hubs showed the same pattern as that for the other hubs. Two other hubs were excluded; one in South Africa that was inactive and one in Uganda, whose members did not respond.

**Table 10:** Active leadership hub members’ self-assessed changes in capacity

Capacity dimension	Jamaica (*n* = 11, three hubs)		Kenya (*n* = 10, two hubs^a^)		Uganda (*n* = 4, one hub^a^)		South Africa (*n* = 9, two hubs)		All countries (*n* = 34, eight hubs)				
	Pre *μ*(SD)	Post *μ*(SD)	Pre *μ*(SD)	Post *μ*(SD)	Pre *μ*(SD)	Post *μ*(SD)	Pre *μ*(SD)	Post *μ*(SD)	Pre *μ*(SD)	Post *μ*(SD)	*t*	Df	*p* value^b^
1 Appraising existing evidence and identifying gaps	3.45 (1.72)	8.00 (0.60)	4.10 (0.94)	7.20 (0.98)	2.50 (1.12)	7.50 (2.18)	3.56 (1.34)	7.22 (1.40)	3.56 (1.44)	7.50 (1.27)	20.40	33	<0.00001
2 Initiating and undertaking an evaluation project	3.36 (2.14)	7.82 (0.72)	3.30 (1.27)	8.10 (0.83)	2.00 (0.71)	7.00 (1.87)	2.89 (1.29)	7.22 (1.69)	3.06 (1.63)	7.65 (1.30)	23.07	33	<0.00001
3 Ability to disseminate findings	4.55 (2.57)	8.18 (0.94)	4.80 (1.25)	7.90 (1.04)	3.25 (1.48)	7.25 (1.48)	4.44 (1.64)	8.33 (1.56)	4.44 (1.94)	8.03 (1.27)	12.66	33	<0.00001
4 Valuing policy relevance and access	3.91 (2.35)	8.36 (1.43)	4.00 (1.41)	7.70 (1.19)	2.25 (1.09)	5.25 (1.79)	3.22 (1.13)	8.11 (1.66)	3.56 (1.79)	7.74 (1.75)	14.31	33	<0.00001
5 Confidence to communicate to decision-makers	4.36 (2.46)	8.55 (0.89)	4.90 (1.30)	8.50 (1.28)	3.25 (1.64)	7.00 (1.41)	3.33 (1.70)	8.33 (1.76)	4.12 (2.00)	8.29 (1.43)	14.78	33	<0.00001
6 Valuing contributions from people in different roles and levels	5.00 (2.41)	8.91 (0.79)	6.10 (1.92)	9.00 (1.26)	3.25 (1.48)	8.00 (1.22)	3.67 (1.56)	8.00 (1.33)	4.76 (2.24)	8.59 (1.24)	12.03	33	<0.00001
7 Leadership and team skills to improve the health system	4.64 (2.19)	8.64 (1.07)	6.20 (1.33)	9.10 (0.70)	4.00 (1.73)	8.75 (0.83)	4.56 (0.96)	8.89 (1.20)	5.00 (1.81)	8.85 (1.00)	17.10	33	<0.00001

^a^Data from two hubs (one in Kenya, one in Uganda) were excluded from significance testing, as only aggregated data, rather than individual data were provided by the RA. Competency scores from these two hubs showed the same pattern as that for the other hubs

^b^A paired *t* test was used to determine the significance of pre versus post differences

## Discussion

We were able to set up, train and sustain leadership hubs in all countries over the 4-year project. However, limited changes in outcomes were observed in the study districts. We consider reasons for these overall findings and differences across countries, in the sections below.

### Baseline comparisons

At baseline, our study findings showed that all study countries had some significant gaps in evidence-informed HIV care, quality assurance initiatives and workplace policies. Family care, referrals and information exchange between health care settings were the most common gaps reported in all countries. It is not surprising that in busy and understaffed clinics and in-patient units, family-related issues get less attention. Involving family members in patient care is compounded by confidentiality issues related to HIV disclosure to patients’ partners, and concerns about stigmatisation [9, 88]. Nurses may have assumed that family dimensions of HIV care were being addressed through voluntary counselling and testing programmes, or by other members of the health care team. There are also country-specific reasons for these findings. For instance, nurses working in Jamaica’s health centres have little interaction with HIV patients after they are diagnosed, as follow-up care is provided by doctors and adherence counsellors.

There were gaps in workplace policies and quality assurance programmes as well. A number of these mirrored the gaps observed for clinical care. There was also more variability across countries with respect to these policies and programmes. These reflect, in part, considerable differences in the HIV policy context [89] and socio-political influences such as legislation and norms regarding homosexuality.

### Pre-post changes in clinical practices, workplace policies and quality assurance

Despite significant improvements in the self-rated capacity of hub members in all countries, there were only small, although statistically significant, pre-post improvements in workplace policies and quality assurance in Jamaica (pre-post, intervention versus control group comparisons) and modest improvements in clinical practices, workplace policies and quality assurance in South Africa (pre-post comparisons). There were small but statistically significant improvements in pre-post scores for nurses stigmatising patients in Jamaica, Kenya and South Africa and for nurses being stigmatised in Kenya, but no statistical adjustment was made for either multiple testing or clustering effects. Multivariate analysis models for Jamaica and Kenya that adjusted for differences across WHO institution levels yielded non-significant results. In Uganda, improvements in the control group for both stigma and quality assurance exceeded those in the intervention groups. The biggest pre-post changes in the intervention groups were seen in South Africa. However, there was no control group in this country.

### Co-interventions

The improvement in stigma scores across all countries (nurses’ reports of both stigmatising and being stigmatised), in both intervention and control groups, suggests a trend towards less HIV-related stigma. Stigma reduction has been a priority for many HIV programmes. In Jamaica, for example, an island-wide stigma reduction campaign was introduced during the study period by the Pan Caribbean Partnership against AIDS [90]. In Kenya, Uganda and South Africa, numerous professional development training programmes targeting service delivery issues including voluntary testing and counselling, stigma, access to HIV care and integrated models of care were offered by Ministries, development aid and non-governmental organisations during the study period [4, 12, 19, 20, 31, 34, 42, 45, 47]. Changes in national level policies on access to HIV care were also evident during the study period with some of the biggest change taking place in South Africa [83]. We did not systematically track these co-interventions. It seems likely that these other interventions may have led to some of the improvements observed, particularly in South Africa.

### Hub intervention

Since trust among partners is a critical prerequisite for effective PAR [91], this may have influenced how quickly the intervention took hold and what activities were prioritised by hubs. Pre-existing relationships between the institutions where research project leads for the hub intervention were employed and the study districts varied substantially.

Our process evaluation of the hubs highlights some strengths of the intervention. The hubs comprised a corps of nurses and health system stakeholders committed to improving HIV prevention and care-related services and policies. Hub members in all districts reported capacity improvements. They gained introductory skills in research, evaluation and influencing workplace policies. However, our experience working with hub members to develop and refine their evaluation projects suggests that in some cases, their self-reported confidence in using these skills may have outstripped their actual ability to use them independently.

A core group of hub members remained engaged throughout the 4-year project period and 11 of the 12 hubs remained active at the end of the programme; 71.7 % of those who were still in the hub at the end of the study had been involved in the hub since the outset. Country directors were able to oversee the recruitment of hub members and replace those who left early, suggesting that the opportunity to participate as a leadership hub member was valued and hubs as an entity were sustained. The substantial proportion of unremunerated time hub members spent attending training and meetings, and developing and implementing both their action plans and evaluation projects also indicates that this opportunity was viewed positively.

Overall, despite these apparent strengths of the leadership hub intervention, district-wide improvements in practice and policy outcomes were not seen. There are two major plausible explanations for these results: the strength and intensity of the intervention was inadequate, and/or outcome measures were not sensitive to intervention effects.

### Intervention strength and intensity

Several factors diluted the potential strength of the hub intervention. Hub activities were significantly disrupted in areas where there were more acute shortages of health care providers and challenging socio-political influences. In both Kenya and Uganda, for example, major reorganisations of their health system led to the redeployment of many health workers, including some hub members. This led to some loss of momentum among hubs as health workers became demoralised as a result of these larger system changes, particularly in Uganda and Kenya.

There were delays in getting baseline data findings to the hubs for their input and feedback, and this may have initially discouraged members. Because we piggy-backed capacity building of country RAs with analysis, results were shared more slowly than initially planned and hubs were sometimes kept waiting for study findings [77]. This may have contributed to some of the unevenness we observed in the content of the action plans across countries and the initial inclusion of some non-HIV-related activities in some of the earlier plans.

While we attempted to ensure links with the formal system by establishing national advisory committees within three countries, requiring that hub members were employed in the study districts and asking hub members to seek their employers’ permission to participate in hub activities, the hubs as an entity were not a recognised formal structure of the health system. Research highlights the importance of integrated governance structures, formal decision-making authority and accountability chains within the system as dimensions of committees that can stimulate wide-scale system change [92–94]. However, these dimensions were missing from our hubs.

Hub members found their work priorities and responsibilities competing with hub time commitments. Furthermore, hub members were not paid by our programme, an approach that was different from the more common practice of non-governmental organisations in the study countries, which normally pay a training allowance or replacement wages to the employee’s institution. Coupled with the fact that leadership hubs were not a formal part of the health care system, this made it difficult for hub members to negotiate release time with their employers. This was even more challenging when districts were dealing with urgent matters such as disease outbreaks (e.g., cholera, H1N1), local natural disasters, national health campaigns, periods of civil unrest and labour disruptions. All of these put extra demands on the scarce human resources available.

Hub members set priorities for two core activities—preparing an action plan and developing an evaluation project. The former posed challenges because the hubs were informal structures, without any direct accountability within the system. This left no formal mechanism for approving their action plans and led to questions about what they were authorised to do. In contrast, the development of evaluation plans provided a turning point for the hubs. Members had to seek administrative approval of participating institutions where they decided to undertake the work. This gave a formal accountability mechanism to provide feedback and recommendations for action. However, in most instances, evaluation projects were completed just a few months before follow-up data for the study were gathered and involved a subset of sampled organisations in the intervention districts. In South Africa, the two active hubs carried out a joint project which involved a literature review and subsequent development of an assessment instrument. Actual implementation of this assessment instrument (to improve continuity of care for HIV patients) was planned for after the project period. Therefore, changes seen in South Africa are not likely due to their evaluation project.

### Sensitivity of outcome measures

HIV care outcome measures for the study were chosen in advance of establishing the hubs. Therefore, they did not reflect the specific focus of the hub action plans and evaluation projects. Since the interventions were hub-driven, similar to a “community-driven” intervention [95], this put our PAR approach at odds with our quasi-experimental design and outcome measures. Yet, the quantitative baseline measures were a critical input into the PAR process. Baseline data comprised an essential set of local and evidence-informed HIV health care indicators that were specific to nursing practice, which hub members considered as they developed their action plans and decided on a focus for their evaluation projects.

Action plans developed by hubs were diffuse. Although evaluation projects were focused, they did not nearly cover the breadth of outcomes assessed. We did not impose a requirement for hub activities to be undertaken in the randomly sampled institutions where study data was being collected and more than half of the hub members came from health facilities or other institutions that were not part of the study sample. Furthermore, not all study institutions had hub members. Thus, we had a district-wide, health system improvement orientation to our sampling and measurement, while hubs chose a more targeted approach to their implementation of action plans and evaluation projects, which did not involve all sampled organisations. In retrospect, we set the bar extremely high in terms of expected outcomes, as hub members were neither working to create changes across the full spectrum of study measures nor representing or targeting all sampled study institutions.

### Research limitations

There were several limitations of the research design. One of these was planned, while others emerged during the study. We did not have a control group in South Africa. This was due to budgetary constraints. A control group in this country would have helped our interpretation of the pre-post improvements we observed. Uganda experienced the biggest challenges in data collection with some notable gaps in the institutions from which data were collected during follow-up. There were several reasons for this including unanticipated changes in research assistants who had to be retrained and some other internal difficulties in that country. This further limits our interpretation of findings from Uganda and meant that we could not include that country in the multivariate analysis. Working across the four countries was a logistical challenge. We had a strong governance structure for our project which helped us operationalise the work but day-to-day communication when problems arose was difficult due to limited internet and challenging phone connections (particularly in Kenya and Uganda) at the time of the study. Our annual face-to-face meetings were critical to the work of the project and both research assistants and study investigators attended these meetings. However, providing timely support for colleagues when issues arose during field work was more challenging.

### Intervention design challenges

In retrospect, this project had three main intervention design challenges. First, leadership hubs did not exist within the formal decision-making and accountability structure of the health system, which would otherwise have provided oversight, support and recognition. Second, we did not give preference to data that could be rapidly and iteratively collected, such as data from district health information systems as reported in some other large-scale evaluations of HIV programmes [96]. This would have complemented data collected by our team and provided timely, ongoing feedback to hub members on progress made. Third, we did not adequately extend the membership reach of the hubs nor did we actively recruit a critical mass of hub members in larger health care institutions. Finally, neither hub members nor their home organisations received any remuneration for participation in hub activities, and we did not offer funding for activities planned as part of hub action plans. Providing some remuneration would have allowed for more rapid implementation of hub activities, although this would fail to address either the underlying constraint of limited human resources or the informal nature of the hubs.

### Future research

Although our capacity measures did capture some characteristics of leadership, we did not use a comprehensive measure of leadership among hub members. Other leadership attributes such as interpersonal relationships, future vision and the ability to manage change [97] would be useful to include in future studies. We only measured self-assessed perceptions of capacity among hub members at the end of the project. Repeated measures would allow further delineation of the pathways through which leadership characteristics develop and exert change.

Synchronising hub initiatives with other programmes targeting HIV could help leverage conditions for change and would be a useful area for future inquiry. During the final phase of the study, some of this synchronisation started to take place but we did not actively catalyse these alignment opportunities.

Although we experienced a number of implementation challenges reflecting the diversity of contexts in which the study was undertaken, we think that comparative research across district and country sites remains important. It is through such comparative studies that insights can be gleaned on how interventions interact with dynamic health care systems and how interventions need to be adapted and tailored to local conditions. In this study, action plans and evaluation projects reflected local concerns and the situational analysis undertaken through the PAR process. We provide a more detailed description of how these contexts impacted on the PAR processes and the experience of leadership hub members within the study in another paper [76].

### Contribution to new knowledge

This is the only study we are aware of that has attempted to link a PAR and leadership-oriented intervention to outcomes at the district health system level. The study highlights promising elements of an intervention to improve evidence-informed nursing care practices and policies by strengthening nurses’ leadership and policy engagement capacity but illustrates the challenges of achieving health system impact.

### Limitations

Our study had several limitations. We were unable to include control districts in South Africa due to budgetary constraints. For some indicators and especially in Kenya, baseline scores on some clinical practices and workplace policies were quite high, with limited room for improvement.

Hub members provided a self-assessment of their pre and post capacity at the end of the study. Had we assessed their skills at the outset of their involvement on hubs, their baseline results may have been different. We do not know if capacity gains were sustained over time.

We did not include quantitative indicators of management, leadership or governance at the level of the health care institutions, other than a human resources management rapid assessment tool [98]. Results from the latter tool, which was completed by a purposeful sample of respondents, are being written up in a separate manuscript. Some dimensions of governance were examined by leadership hubs in their evaluation projects.

## Conclusions

PAR, with collaboration between nurses and decision-makers, can bring to light gaps in the health care system and identify ways to improve clinical practice and care. Leadership hubs comprising people capable of and committed to change and provided with capacity building and mentorship can collectively identify issues and act on strategies that may improve practice and policy. It is apparent that targeted change strategies are a more realistic short-term expectation of leadership hubs than district-level health system improvements. Funding hub-led evaluation projects created a necessary mechanism for hub reporting, feedback and accountability within the health care system. This type of mechanism is essential for formalised systems’ change processes.

Overall, leadership hubs did not provide the necessary force for nurses to improve HIV care in their districts. If entities such as leadership hubs are to succeed, they must be integrated within district health authorities as participatory policy and practice mechanisms and become part of established formal, legal organisations (such as nursing associations or academic institutions) in order to regularise and sustain them as a means to improve health system performance.

### Disclosures

In 2007, a large multidisciplinary team of researchers and decision-makers from Canada and five LMICs (Barbados, Jamaica, Kenya, Uganda and South Africa) received funding to implement a PAR programme entitled “Strengthening Nurses’ Capacity for HIV Policy Development in sub-Saharan Africa and the Caribbean.” One year after programme funding was received and prior to any data collection, Barbados withdrew from the programme, with the four remaining partner countries continuing [77].

## Abbreviations

AIDS, acquired immune deficiency syndrome; ANOVA, analysis of variance; ART, antiretroviral therapy; CHWs, community health workers; HIV, human immunodeficiency virus; HSD, honestly significant difference; LMICs, low- and middle-income countries; PAR, participatory action research; RAs, research assistants; SD, standard deviation; WHO, World Health Organization

## Additional file

Additional file 1: Table S1. Ethics Approvals & Institutional Letters of Permission. (DOC 34 kb)

## Footnotes

1 No further differentiation is made between nurses and midwives; the term nurse is used for the rest of the article.

2 “Strengthening Nurses’ Capacity in HIV Policy Development in Sub-Saharan Africa and the Caribbean”.

3 District refers to both districts (Kenya, Uganda, South Africa) and parishes (Jamaica).

4 No control group was included in South Africa due to budgetary constraints.

5 The lead project researcher in each country provided direction and day-to-day guidance for all research activities in that country.

6 Level 3 corresponds to health centres; level 4 are district hospitals; and level 5 are provincial and national referral hospitals.

7 To be eligible for enrolment in the study, health centres had to meet the WHO health criteria for staffing, and not just be designated as a health centres by the Ministry of Health.
