# Supplementary material for: The impact of leadership hubs on the uptake of evidence-informed nursing practices and workplace policies for HIV care: a quasi-experimental study in Jamaica, Kenya, Uganda and South Africa
Source: Implement Sci. 2016 Aug 3;11:110. doi: 10.1186/s13012-016-0478-3 (PMC4973110; doi:10.1186/s13012-016-0478-3)
Supplement: Additional file 1: Table S1. — Ethics Approvals & Institutional Letters of Permission. (DOC 34 kb) [file 13012_2016_478_MOESM1_ESM.doc]

**Table S1. Ethics Approvals & Institutional Letters of Permission**

| **Research Ethics Boards** | |
| --- | --- |
| **Country** | **Research Ethics Board** |
| Canada | University of Ottawa |
| Canada | University of Alberta |
| Canada | University of Toronto |
| Canada | University of Lethbridge |
| Canada | Dalhousie University |
| Jamaica | Ministry of Health, Jamaica |
| Jamaica | University of the West Indies, Mona campus |
| Jamaica | Kingston Public Hospital Ethics Committee (SERHA) – KPH/VJH |
| Kenya | Kenya Medical Research Ethics Committee |
| Kenya | Great Lakes University of Kisumu |
| Uganda | Makerere University |
| Uganda | Uganda National Council of Science and Technology |
| Uganda | Mulago Hospital |
| South Africa | North West University |
| South Africa | Northwest Provincial Health Department |
